# Comparative impact of fungal and microbial proteases on the rumen and fecal microbiota composition and nutrient digestibility in Kazakh White Head bulls

**DOI:** 10.14202/vetworld.2025.3536-3544

**Published:** 2025-11-27

**Authors:** Viktoriya Vladimirovna Grechkina, Elena Vladimirovna Sheida, Olga Vilorievna Kvan, Artem Vladimirovich Bykov

**Affiliations:** 1 Federal Scientific Center of Biological Systems and Agrotechnologies of the Russian Academy of Sciences, ul. 9 January, 29, Orenburg, 460000, Russian Federation; 2 Department of Non-Communicable Animal Diseases, Orenburg State Agrarian University, ul. Chelyuskintsev, 18, Orenburg, 460014, Russian Federation; 3 Department of Food Biotechnology, FSBEI HE “Orenburg State University,” 460018, Orenburg, Povedy avenue, 13, Russia; 4 Department of Feeding and Feed Technology Named after S.G. Leushin, Federal Scientific Center for Biological Systems and Agro-Technologies of the Russian Academy of Sciences, 29 9^th^ January Str., Orenburg, 460000, Russia; 5 Department of Food Biotechnology, Doctor of Biological Sciences, Associate Professor, Federal State Budgetary Educational Institution of Higher Education “Orenburg State University”460018, Orenburg, Povedy avenue, 13, Russia

**Keywords:** cattle, fungal protease, microbial protease, next-generation sequencing, nutrient digestibility, rumen microbiota

## Abstract

**Background and Aim:**

Proteases are key enzymes that hydrolyze peptide bonds enhancing the utilization of feed protein, improving nutrient efficiency, and reducing the need for costly protein ingredients. Despite their growing use in animal nutrition, comparative studies between fungal and microbial proteases in ruminants remain scarce. This study aimed to evaluate the influence of fungal and microbial proteases (25 U/g each) on the microbial composition of rumen fluid and feces, and on the chemical composition of digestive contents in Kazakh White Head bulls.

**Materials and Methods:**

Twenty bulls (14–15 months old; 310–320 kg) fitted with rumen fistulas were divided into three groups: a control group and two treatment groups, each receiving a basal diet supplemented with either fungal or microbial protease. Rumen fluid and fecal samples were analyzed for taxonomic profiles using next-generation sequencing (MiSeq, Illumina) of the 16S ribosomal RNA V3–V4 region. Chemical composition (dry matter [DM], crude protein [CP], crude fat [CF], crude fiber, and ash) was determined according to GOST mass fraction of DM 31640, mass fraction of CP 13496.4, mass fraction of CF 13496.15, mass fraction of crude fiber 31675, and mass fraction of crude ash 26226 standards. Statistical analysis was performed using the Mann–Whitney U-test (p ≤ 0.05).

**Results:**

Microbial protease supplementation increased the abundance of beneficial phyla *Bacillota* (70.1%) and *Bacteroidota* (19.5%) in rumen fluid, with a corresponding rise in DM (+6.3%), CF (+9.4%), and CP (+7.9%) relative to control. In feces, *Bacillota* (70.7%) and *Bacteroidota* (15.5%) predominated. No opportunistic pathogens (e.g., *Pseudomonas* and *Sutterella*) were detected in the microbial protease group, indicating improved microbial balance and intestinal protection. Fungal protease exerted milder effects, with modest increases in nutrient fractions.

**Conclusion:**

Microbial protease was more effective than fungal protease in optimizing rumen microbiota and enhancing nutrient digestibility in bulls. Its use may support environmentally sustainable livestock production by reducing nitrogen excretion and dependence on high-protein feed ingredients. These findings provide a scientific basis for breed-adapted enzymatic feeding strategies in ruminants.

## INTRODUCTION

Incorporating enzymes into animal feed enhances the digestion of feed components that animals are otherwise unable to efficiently degrade. Their inclusion allows the use of low-cost raw materials containing antinutritional factors without compromising animal health or productivity [1]. Among these, proteases are gaining increasing attention in animal nutrition worldwide, and their global market is expected to expand substantially in the coming years [2].

The composition of the diet has a strong influence on the structure and diversity of the gastrointestinal microbiota in farm animals. Nutritional imbalances or feeding errors can disrupt microbial homeostasis, leading to dysbiosis, reduced productivity, and increased susceptibility to disease [3]. Conventionally, microbiological methods have been employed to study rumen microflora; however, these approaches are limited by the difficulty of accurately quantifying microorganisms grown on selective media. Advances in molecular genetics now enable culture-independent profiling of microbial diversity, providing a more comprehensive and precise understanding of rumen microbial ecology [4].

Proteases represent the largest segment of the global industrial enzyme market and are widely used in animal feed formulations [5]. They compensate for the deficiency of endogenous enzymes in young animals and poultry whose digestive systems are still developing [6]. Beyond nutrition, proteases exhibit therapeutic properties and are utilized in the treatment of diseases affecting the respiratory, cardiovascular, gastrointestinal, and integumentary systems, as well as ulcers and inflammatory conditions [7].

These enzymes are derived from diverse biological sources, including animal tissues (e.g., calf stomach), plant tissues (e.g., pineapple, fig, and papaya), and microorganisms such as *Bacillus* and *Pseudomonas* species [8]. However, enzyme extraction from animal and plant materials is constrained by ethical concerns, environmental impact, and low yield. Consequently, microbial enzymes have become preferred due to their high productivity, scalability, and broad biochemical versatility [9].

Notably, the dietary use of proteases can reduce ammonia emissions in livestock facilities, improving both animal welfare and the surrounding environment [10]. Overall, enzymes enhance the digestion and absorption of nutrients, mitigate the effects of antinutritional compounds, and supplement the immature enzymatic systems of young animals, thereby promoting optimal growth and health [11].

Although proteases have long been recognized for their ability to improve feed digestibility and nutrient utilization in monogastric animals, their effects on the rumen microbiome of ruminants remain insufficiently characterized. Previous studies have largely focused on enzyme efficacy in poultry and swine, emphasizing growth performance and amino acid digestibility rather than microbial ecology. Moreover, most available data pertain to single-source or plant-based proteases, while comparative analyses between fungal and microbial proteases in ruminant nutrition are scarce. In particular, limited attention has been given to how these enzyme sources alter the taxonomic composition of rumen and fecal microbiota, which plays a critical role in nutrient metabolism, immune modulation, and feed efficiency. Few studies have integrated next-generation sequencing (NGS) to describe these microbial shifts in response to enzyme supplementation. Likewise, the relationship between enzymatic hydrolysis, microbial community structure, and the chemical composition of rumen contents has not been comprehensively explored. In addition, little information exists regarding the Kazakh White Head breed, a locally adapted beef cattle genotype whose rumen microbial ecosystem and enzymatic responses remain poorly documented. Understanding the interaction between enzyme type and microbial ecology in such native breeds is crucial for designing sustainable, breed-adapted feeding systems that minimize nitrogen excretion and environmental emissions.

This study aimed to compare the effects of fungal and microbial protease supplementation on the chemical composition and microbiota of rumen fluid and feces in Kazakh White Head bulls. Specifically, it sought to:

1. Assess how fungal and microbial proteases influence the diversity and relative abundance of bacterial taxa in the rumen and rectum using NGS-based 16S ribosomal RNA (16S rRNA) sequencing.
2. Determine the corresponding changes in the chemical composition (dry matter [DM], crude protein [CP], crude fat [CF], crude fiber, and ash) of rumen fluid and feces as indicators of feed digestibility.
3. Identify whether microbial protease confers superior effects on microbial balance, nutrient absorption, and reduction of opportunistic pathogens compared with fungal protease.
4. Provide evidence supporting the use of microbial protease as a sustainable nutritional strategy to improve feed efficiency and reduce nitrogen emissions in beef production.

## MATERIALS AND METHODS

### Ethical approval

All animal procedures and experimental protocols were performed in accordance with the ethical standards and regulatory acts governing animal welfare. The study complied with the Model Law of the Interparliamentary Assembly of the Member Nations of the Commonwealth of Independent States “On the Treatment of Animals,” Article 20 (Resolution No. 29-17, October 31, 2007). The study was approved by Meeting of the Bioethics Commission of the Federal State Budgetary Scientific Institution (FSC BST RAS Minutes No. 5 of 12.23.24). All efforts were made to minimize animal discomfort and reduce the number of animals used in the study. The experimental protocol also adhered to the Guidelines for Working with Laboratory Animals available at http://fncbst.ru/?page_id=3553.

### Study period and location

The experiment was conducted from May to July 2025 at the Department of Farm Animal Nutrition and Feed Technology named after Prof. S. G. Leushin, Federal State Budgetary Scientific Institution “Federal Scientific Center for Biological Systems and Agricultural Technologies of the Russian Academy of Sciences” (Accreditation Certificate No. PA.RU21PF59, December 2, 2015), Orenburg, Russian Federation.

### Experimental animals and diets

Healthy Kazakh White-Headed bull calves (n = 30; average body weight = 310–320 kg; age = 14–15 months) fitted with chronic rumen fistulas (ANKOM, USA) were used in the experiment. Animals were divided into three groups (n = 10 per group) using the pair-analog method:

- Control group – basal diet without enzyme supplementation
- Fungal protease group – basal diet supplemented with 25 U/g alkaline fungal protease
- Microbial protease group – basal diet supplemented with 25 U/g microbial protease.

All animals were fed a balanced diet following the recommendations of Kalashnikov *et al*. [12]. The diet consisted of mixed hay (47.4%), legume hay (32.6%), grain mixture (19.0%), and mineral supplements (1.0%). The chemical composition of the diet (% of DM) was as follows: DM 94.68, CP 5.0, crude fiber 28.0, neutral detergent fiber 6.3, acid detergent fiber 4.6 (hemicellulose 1.65), CF 2.73, organic matter 93.4, calcium 0.51, phosphorus 0.37, crude ash 1.28, and nitrogen-free extract 53.8. Animals were provided with clean drinking water twice daily.

### Enzyme preparations

- Fungal alkaline protease (protozyme S): A dry enzyme preparation that hydrolyzes proteins into peptides and amino acids (Biopreparat, Voronezh, Russia); enzyme activity = 50,000 U/g.
- Microbial protease (protozyme): A dry preparation derived from *Bacillus licheniformis* through non-genetically modified fermentation, followed by purification and concentration (EdaProf, Moscow, Russia); enzyme activity = 50,000 U/g.

Enzymes were incorporated into feed by stepwise mixing with fillers or finely ground concentrate to ensure uniform distribution. This approach also prevented possible inactivation due to pH or temperature differences among enzyme types. The preparations did not chemically interact with feed ingredients and remained stable during storage for up to 24 months below 25°C. Their acid-resistant coating preserved enzymatic activity in the gastric environment.

### Sampling procedures

Rumen fluid was collected 12 h after feeding through the fistula (diameter = 80 mm) using a sterile rubber hose (200 cm × 40 mm outer diameter) into a 3-L thermos flask. Samples were transported to the laboratory within 30 min. Feed digestion time ranged from 12–14 h, depending on substrate characteristics.

Fecal samples (10 g per animal) were collected directly from the rectum, placed in sterile glass containers with tight-fitting lids, and transported to the laboratory within 15–20 min after collection.

### Microbiome analysis

The taxonomic profile of rumen and fecal microbiota was analyzed at the Biotrof+ LLC Molecular Genetics Laboratory (St. Petersburg, Russia) using NGS of the *16S rRNA* gene.

- DNA extraction: Genomic DNA was isolated using the Genomic DNA Purification Kit (Thermo Fisher Scientific, USA).
- Amplification: The V3–V4 region of the *16S rRNA* gene was amplified with forward primer 5′-TC GTCGGCAGCGTCAGATGTGTATAAGAGACAGCCTACGGGNGGCWGCAG-3′ and reverse primer 5′-GTCTCGTGGG CTCGGAGATGTGTATAAGAGACAGGACTACHVGGGTATCTAATCC-3′. PCR conditions: 95°C for 3 min; 25 cycles of 95°C for 30 s, 55°C for 30 s, 72°C for 30 s; final elongation = 72°C for 5 min.
- Sequencing: Libraries were prepared with the Nextera XT Index Kit (Illumina, USA and purified with Agencourt AMPure XP (Beckman Coulter, USA). Sequencing was performed on the MiSeq platform using the MiSeq Reagent Kit v2 500 cycles (Illumina). Library quality was assessed with the High Sensitivity DNA Kit (Biotium, USA) on an Agilent 2100 Bioanalyzer (Agilent Technologies, USA).
- Bioinformatics: Sequences (median >250 bp) were processed and classified using PyNAST software (https://directory.fsf.org/wiki/PyNAST) with the UNITE fungal database for taxonomic assignment.

### Chemical analysis

Rumen fluid and fecal samples were analyzed for chemical composition (%):

- DM – GOST 31640
- CP – GOST 13496.4
- CF – GOST 13496.15
- Crude fiber – GOST 31675
- Crude ash – GOST 26226.

### Statistical analysis

Data were processed using the Statistical Package for the Social Sciences Statistics 20 (IBM Corp., NY, USA). Mean values (M), standard deviations, and standard errors were calculated. Group differences were analyzed using the non-parametric Mann–Whitney U-test, followed by Dunn’s *post hoc* comparison. Differences were considered statistically significant at p ≤ 0.05 and p ≤ 0.01.

## RESULTS

### Microbial composition of rumen and rectum

**Figure 1:**
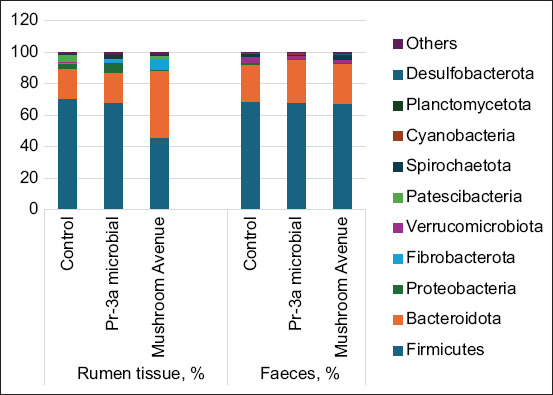
Composition of the microbiota of rumen fluid and fecal samples from calves at the bacterial phyla and superphyla levels (based on next-generation sequencing of *16S ribosomal RNA* gene amplicons).

The microbiome of the rumen fluid and rectum of bulls in all experimental groups comprised 20 bacterial phyla and superphyla. The dominant phyla were *Bacillota*, *Bacteroidota*, and Pseudomonadota. Figure 1 illustrates the ten most common phyla based on average group values.

#### Dominant bacterial phyla

*Bacillota* bacteria predominated in the feces of all experimental groups, accounting for up to 70% of the total population, with the lowest proportion (45%) observed in the rumen fluid of bulls supplemented with fungal protease. Overall, the relative abundance of microbiota in the feces was highest for *Bacillota* (70.7%), followed by *Bacteroidota* (15.5%) and *Verrucomicrobiota* (6.1%).

#### Influence of protease supplementation

The rumen (scar) fluid contained a high proportion of *Bacillota* bacteria, 70.1% in animals receiving microbial protease and 45.1% in those supplemented with fungal protease. The proportion of *Bacteroidota* tended to increase in the microbial protease group compared with the control group, showing a 19.5% higher average abundance.

A notable abundance of the Fibrobacterota taxon (5%) was detected in the rumen fluid of the group receiving 5% fungal protease supplementation. This taxon was absent in the fecal microbiota of both the control and experimental groups. Furthermore, *Pseudomonadota* were more numerous in rumen fluid samples from animals supplemented with microbial protease (6.05 ± 0.12%) than in the control group.

### Microbial community at the family level

**Figure 2:**
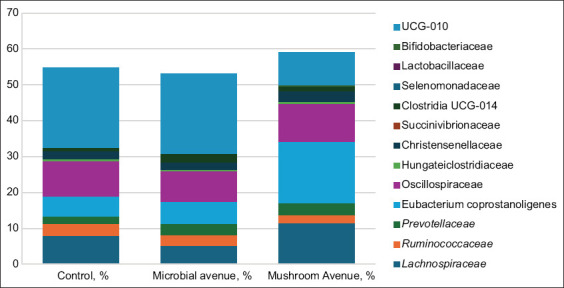
Main families of the rectal microbiome of calves (based on next-generation sequencing of *16S ribosomal RNA* gene amplicons).

A closer examination of microbial composition down to the family level revealed a similar overall pattern across groups. Figure 2 presents the most represented families in the rectal microbiota and their average values per group.

#### Differentially abundant families

Out of 118 families identified, 13 showed significant differences (p < 0.05) compared with the control group. Cellulolytic families such as *Lachnospiraceae* and *Ruminococcaceae* were more abundant in the control and fungal protease groups, representing 24.4% ± 9.1% and 10.9% ± 8.8% of the microbiota, respectively. The *Prevotellaceae* family, which also includes cellulolytic species, increased by 12.3% in the rectal microbiota of animals receiving fungal protease relative to controls, comprising 2%–3% of total bacterial families.

#### Dominant families in rumen fluid

According to Table 1, the dominant families common to all rumen fluid samples were *Prevotellaceae* and *Lachnospiraceae*. The abundance of other taxa varied depending on dietary treatment. In the control group, *Ruminococcaceae* also ranked among the most numerous families, whereas their proportions declined in protease-treated groups.

**Table 1:** Average content of the most represented families in the rumen fluid of the experimental groups, (m ± standard), %.

Family	Control	Microbial protease	Fungal protease
*Lachnospiraceae*	24.3 ± 9.1	26.4 ± 1.4*	23.7 ± 1.8
*Prevotellaceae*	10.9 ± 4.6	10.4 ± 1.2	21.5 ± 3.1
*Ruminococcaceae*	14.6 ± 8.8	11.2 ± 4.1	6.2 ± 1.9
*Oscillospiraceae*	3.8 ± 3.4	12.8 ± 1.6	4.7 ± 1.7
*Rikenellaceae*	4.1 ± 2.0	6.6 ± 1.4*	5.9 ± 0.2
*Clostridia UCG-014*	2.8 ± 1.4	8.4 ± 1.2	9.5 ± 2.5
*Succinivibrionaceae*	1.9 ± 2.4	5.9 ± 1.4	2.3 ± 0.4
*Christensenellaceae*	1.9 ± 1.7	5.8 ± 1.2	1.7 ± 0.1
*Bacteroidales RF16*	1.5 ± 1.3	0.8 ± 0.9	6.0 ± 0.2
*Acholeplasmataceae*	3.7 ± 3.2	1.3 ± 0.2	4.9 ± 0.01*
Others	30.0 ± 5.9	19.9 ± 2.1	24.0 ± 0.4

* p ≤ 0.05 when compared with the control group.

The proportion of *Lachnospiraceae* in rumen fluid microbiota differed by 2.1% between the control and microbial protease groups, with a 2.5% decrease recorded in the fungal protease group. Specifically, *Lachnospiraceae* abundance was 0.6% (p < 0.05) lower in the microbial protease group than in controls, while *Prevotellaceae* abundance increased by 49.3% in the fungal protease group relative to controls.

### Detection of pathogenic and opportunistic bacteria

No opportunistic pathogens or undesirable microorganisms were detected in either the rumen fluid or feces of animals receiving microbial protease supplementation, suggesting enhanced microbial stability and host protection (Table 2). In contrast, the control group exhibited the presence of opportunistic microorganisms, primarily *Actinobacteria* and *Pseudomonas*, including potential causative agents of actinomycosis.

**Table 2:** Average number of bacteria from the families represented in scar tissue fluid and calf feces, %.

Family	Rumen fluid			Feces		
	
	Control	Microbial protease	Fungal protease	Control	Microbial protease	Fungal protease
*Pseudomonas*	0.053	0	0	0.057	0	0.020
*Streptococcus*	0	0	0	0	0	0
*Acinetobacter*	0.271	0	0	0.104	0	0
*Staphylococcus*	0	0	0	0.75	0	0.28
*Stenotrophomonas*	0	0	0	0	0	0
*Sutterella*	0	0	0	0.010	0	0

In the group fed fungal protease, *Pseudomonas* bacteria were detected at a minimal level (0.02%). The genus *Sutterella*, which is associated with inflammatory bowel disease, was observed only in the feces of control animals, accounting for 0.010% of the total bacterial community.

### Chemical composition and digestibility

The digestibility of nutrients is a critical determinant of digestive system efficiency and overall feed nutritional value (Table 3). Microbial protease supplementation improved the chemical composition of rumen fluid, increasing DM by 6.3%, CF by 9.4%, and CP by 7.9% (p < 0.05) compared with controls.

**Table 3:** Chemical composition of scar tissue fluid and feces when using exoenzymes, %.

Indicators	Rumen fluid			Feces		
	
	Control	Microbial protease	Fungal protease	Control	Microbial protease	Fungal protease
Mass fraction of the dry matter	65.71 ± 2.13	70.12 ± 2.22*	68.91 ± 1.89	66.22 ± 2.02	68.94 ± 2.24	68.82 ± 2.11
Mass fraction of the crude fat	74.93 ± 1.89	82.73 ± 2.14	81.92 ± 2.65	75.14 ± 1.42	77.85 ± 2.75	77.24 ± 1.87
Mass fraction of the crude protein	57.72 ± 2.14	62.71 ± 2.22*	62.94 ± 1.99	58.03 ± 2.14	60.83 ± 1.87	60.23 ± 2.11
Mass fraction of the crude fiber	51.24 ± 2.36	55.90 ± 2.15*	54.52 ± 1.99	52.17 ± 2.11	54.26 ± 2.02	54.51 ± 1.89
Mass fraction of the crude ash	73.41 ± 3.21	77.32 ± 2.87	76.63 ± 2.45	75.13 ± 3.46	82.17 ± 2.68*	81.32 ± 2.87

* p ≤ 0.05 when compared with the control group.

#### Effect of fungal protease

Fungal protease supplementation produced a less pronounced effect, with slight increases in DM (3.8%), CP (3.7%), and crude fiber (4.4%) compared with controls. Differences in microbial diversity may have influenced variations in nutrient digestibility among groups.

#### Broader implications of protease supplementation

Recent research suggests that the benefits of protease enzymes extend beyond protein hydrolysis to include “extra-protein” effects—such as improved digestibility of fats and starches, stabilization of the intestinal microbiome, enhanced herd uniformity, and reduced nitrogen excretion in feces. While these effects are secondary to the enzymes’ primary function, they have become key factors influencing the modern use of proteases in ruminant nutrition.

## DISCUSSION

### Dominant microbial phyla and their functional role

In the feces of the experimental groups, *Bacillota* (70.7%) was the most dominant phylum, followed by *Bacteroidota* (15.5%) and *Verrucomicrobiota* (6.1%). These bacterial groups are primarily involved in the fermentation of complex polysaccharides in feed and the production of short-chain fatty acids (SCFAs), which serve as a major energy source for ruminants. The rumen microbiota plays a pivotal role in breaking down plant-derived materials into energy-rich compounds that sustain host metabolism and productivity [13].

### Differences between rumen and intestinal microbiota

The microbial community of the rectum differs substantially from that of the rumen, reflecting adaptation to distinct physiological environments. While the rumen microbiota specializes in the degradation of fibrous carbohydrates, the intestinal microbiota is composed of bacteria better suited for nutrient absorption and secondary fermentation processes. The composition of intestinal bacteria is influenced by niche-specific factors, such as nutrient availability, pH gradients, and host-microbe interactions that govern microbial colonization dynamics [14].

### Effect of protease supplementation on microbial structure and feed efficiency

Previous studies have demonstrated that protease supplementation can beneficially modulate the gut microbiota and improve nutrient utilization. According to Bernardeau *et al*. [15], the inclusion of protease in feed formulations can reduce dietary protein and amino acid requirements by 4%–6%, enabling a lower dependence on expensive protein ingredients without compromising performance.

Experimental work with alkaline protease derived from *Bacillus licheniformis* has shown its ability to restructure the intestinal microbial community, enhancing populations that produce beneficial metabolites and improving gut health. Proteases hydrolyze complex proteins into amino acids and peptides, thereby enhancing protein digestibility and nutrient absorption efficiency. As a result, feed formulations containing protease can maintain nutritional adequacy while reducing the inclusion of costly ingredients such as soybean meal.

### Role of commensal and opportunistic bacteria in gut health

The genus *Sutterella*, identified in the control and fungal protease groups, represents a commensal bacterium that may become pathogenic under conditions of dysbiosis or compromised immunity. Although *Sutterella* contributes to immune system maturation, its overgrowth or translocation may predispose cattle to inflammatory or autoimmune conditions [16].

Opportunistic microorganisms, such as *Actinobacteria* and *Pseudomonas*, typically coexist harmlessly within the host but may cause disease when the immune defenses weaken. Disruption of the intestinal mucosal barrier can facilitate microbial translocation, eliciting systemic immune responses through immunoglobulin G secretion. In certain cases, cross-reactivity between microbial antigens and host autoantigens can trigger autoimmune disorders [17].

Notably, in the present study, no pathogenic microorganisms were detected in the rumen or fecal samples of bulls receiving microbial protease, suggesting that the enzyme exerted a protective effect on intestinal microbial ecology. This implies that microbial protease supplementation not only enhances digestion but also supports gut stability and host immunity, thereby reducing reliance on protein-rich feed additives.

### Mechanistic insights into enzymatic modulation of rumen function

Recent findings by Zhao *et al*. [18] further support the hypothesis that the inclusion of bacterial and fungal proteases significantly alters rumen metabolic activity and enhances nutrient assimilation in ruminants. Enzymes promote more efficient hydrolysis of feed components, improving fermentation balance and feed conversion efficiency.

The effectiveness of proteases in ruminant nutrition depends on several factors, including the enzyme source, activity spectrum, substrate specificity, and the rumen environment. Therefore, continued research on the mechanisms of enzyme-microbiota interaction is essential for developing sustainable enzymatic feeding strategies that maximize nutrient absorption and improve livestock productivity across different species and production systems.

## CONCLUSION

The present study demonstrated that microbial protease supplementation was more effective than fungal protease in modulating rumen fermentation, improving nutrient digestibility, and enhancing microbial balance in Kazakh White Head bulls. Supplementation with microbial protease resulted in a 6.3% increase in DM, a 9.4% increase in CF, and a 7.9% increase in CP content in the rumen fluid compared with the control. This effect corresponded with a higher abundance of beneficial phyla, particularly *Bacillota* (70.1%) and *Bacteroidota* (19.5%), which are associated with efficient fiber degradation and SCFAs production. Importantly, no opportunistic pathogens were detected in the microbial protease group, indicating improved intestinal microbial stability and host protection.

From a practical standpoint, these findings suggest that microbial protease can partially replace dietary protein requirements, allowing for the use of lower-cost feed formulations while maintaining productivity. By improving nutrient assimilation and reducing nitrogen excretion, microbial proteases contribute to environmentally sustainable livestock production and better farm air quality. The study also underscores the potential of microbial enzymes as alternatives to chemical feed additives for improving digestion and rumen ecology in beef cattle.

The major strength of this research lies in its use of NGS-based microbiome profiling, which provided high-resolution insights into microbial community shifts at both phylum and family levels. In addition, it is one of the first comparative studies to evaluate fungal versus microbial protease effects in a native cattle breed under controlled feeding conditions.

However, the limitations include a relatively small sample size (n = 10 per group) and a short experimental duration, which may not fully reflect long-term enzyme–microbiota dynamics or production outcomes, such as growth rate and feed conversion efficiency.

Future studies should therefore focus on long-term feeding trials integrating metabolomic and metatranscriptomic analyses to better understand the functional consequences of enzyme-induced microbiome modulation. Moreover, exploring optimal enzyme dosages, combinations with other hydrolases (e.g., amylase and cellulase), and the effects on methane reduction would expand the applicability of protease technology in ruminant nutrition.

Microbial protease supplementation represents a promising, sustainable, and biologically safe strategy for enhancing feed efficiency, maintaining intestinal health, and reducing environmental burden in ruminant production systems. These findings provide a scientific foundation for developing breed-adapted enzyme-based feeding programs that align with modern goals of productivity, profitability, and ecological responsibility.

## DATA AVAILABILITY

The supplementary data can be made available from the corresponding author upon request.

## AUTHORS’ CONTRIBUTIONS

VVG, EVS, OVK, and AVB: Contributed to the conception and design of the study. VVG: Supervised the study and edited the manuscript. EVS: Data and sample collection, laboratory tests, data analysis and interpretation, and drafted and revised the manuscript. OVK and AVB: Laboratory tests, data analysis and interpretation, and revised the manuscript. All authors have read and approved the final version of the manuscript.
